# Long‐term outcomes of stereotactic radiofrequency ablation in hypothalamic hamartomas: A single‐center experience

**DOI:** 10.1111/epi.18660

**Published:** 2025-10-01

**Authors:** Peter Christoph Reinacher, Julia Jacobs, Mukesch Johannes Shah, Theo Demerath, Kathrin Wagner, Victoria San Antonio‐Arce, Horst Urbach, Volker Arnd Coenen, Andreas Schulze‐Bonhage, Kerstin Alexandra Klotz

**Affiliations:** ^1^ Department of Stereotactic and Functional Neurosurgery Medical Center–University of Freiburg, Faculty of Medicine–University of Freiburg Freiburg Germany; ^2^ Fraunhofer Institute for Laser Technology Aachen Germany; ^3^ Department of Neuropediatrics and Muscular Disorders Medical Center–University of Freiburg, Faculty of Medicine–University of Freiburg Freiburg Germany; ^4^ Hotchkiss Brain Institute and Alberta Children's Hospital Research Institute University of Calgary Calgary Alberta Canada; ^5^ Department of Neurosurgery Medical Center–University of Freiburg, Faculty of Medicine–University of Freiburg Freiburg Germany; ^6^ Department of Neuroradiology Medical Center–University of Freiburg, Faculty of Medicine–University of Freiburg Freiburg Germany; ^7^ Epilepsy Center Medical Center–University of Freiburg, Faculty of Medicine–University of Freiburg Freiburg Germany; ^8^ Member of the European Reference Network for rare and complex epilepsies EpiCARE Freiburg Germany; ^9^ Department of Neuropediatrics University Hospital Bonn Bonn Germany

**Keywords:** gelastic seizures, hypothalamic hamartoma, refractory epilepsy, stereotactic radiofrequency coagulation, surgical outcome

## Abstract

**Objective:**

Hypothalamic hamartomas (HHs) lead to refractory epilepsy, and minimally invasive surgical approaches are standard of care for affected patients. Stereotactic radiofrequency thermocoagulation (SRT) is one of the treatment methods recognized to achieve seizure freedom. This study reports surgical outcome from a single center reporting an ablation technique using fewer trajectories than previously reported and assesses the effect of coagulated volume on long‐term seizure freedom.

**Methods:**

Retrospective analysis was made of all patients who underwent SRT at the University of Freiburg between 2016 and 2024 with a follow‐up of ≥12 months. Statistical analysis was made of outcome dependent on type of hamartoma, seizure type, coagulation volume (based on magnetic resonance imaging evaluation), and epilepsy duration.

**Results:**

Forty‐three patients received SRT; 35 (22 children) had >12 months of follow‐up, with a median of 38 months. Nine patients had two SRTs, and two patients had three SRTs. Twelve months after their last SRT, 60% of patients were seizure‐free, 88.6% were free of bilateral tonic–clonic seizures, and 77.1% were free of gelastic seizures (last follow‐up: 54.3% seizure‐free, 88.6% free of bilateral tonic–clonic seizures, 74.3% free of gelastic seizures). There was a significant reduction of antiseizure medication (ASM) postsurgically, with an average number of ASMs of two prior to surgery and one after surgery. After 12 months, 14.3% of patients experienced ongoing but mostly mild surgical complications, with hypothalamic dysfunction being the most common. Coagulation volumes were higher in larger HHs, but no correlation was observed between coagulated volume and seizure freedom or complication rates.

**Significance:**

SRT is a minimally invasive method to successfully treat refractory seizures in patients with HH. Disconnection seems to be more important for successful treatment than volume reduction. Even large HHs can be successfully treated with small coagulation volumes.

**Key points**

- Stereotactic radiofrequency thermocoagulation achieved 60% seizure freedom at 12 months using fewer trajectories than previously reported approaches.
- A total of 88.6% of patients became free of bilateral tonic–clonic seizures, with significant ASM reduction.
- Outcome depends more on effective hamartoma disconnection than on size reduction or coagulated volume.
- Complication rates were low, with mostly mild hypothalamic dysfunction as a persistent complication.

## 1 INTRODUCTION

Hypothalamic hamartoma (HH) syndrome is a rare developmental condition characterized by a deep‐seated hamartoma with varied attachment to the hypothalamus. Affected patients experience a combination of symptoms with a wide range of severity including refractory epilepsy, endocrine abnormalities, encephalopathy, psychiatric comorbidities, and developmental delay.^1^ A hallmark syndrome of HH‐related seizures is gelastic and dacrystic seizures.^2^ Epilepsy in HH often manifests within the first years of life, and gelastic seizures commonly are the first seizure type. Diagnosis is often delayed until further seizures appear that are easier to recognize.^3^ Depending on the population studied, it is estimated that 60%–100% of patients with epilepsy in HH are refractory to antiseizure medications (ASMs), and gelastic seizures are specifically refractory to treatment despite polypharmacy.^4^, ^5^ Therefore, international recommendations suggest early presurgical investigations in all patients affected by HH and epilepsy even prior to failing ASM treatment.^6^

Due to its deep location close to the hypothalamus, open surgical approaches to the HH are difficult and associated with high complication rates. Current standards of care are therefore minimally invasive approaches, including stereotactic radiofrequency thermocoagulation (SRT), laser interstitial thermal therapy (LITT), radiosurgery (gamma‐knife), and robot‐guided stereo‐endoscopic approaches utilizing laser or radiofrequency disconnection techniques. These robotic endoscopic methods allow safe and complete disconnection through single intraparenchymal trajectories with multiple probe placements within the ventricle, offering an alternative to conventional open microsurgical approaches.^7^, ^8^, ^9^ Three recent meta‐analyses including more than 400 patients each suggest that these three methods have similar complication rates, but SRT and LITT have slightly better surgical outcomes in regard to seizure freedom.^10^, ^11^, ^12^ This might also be related to the finding that effects of radiosurgery can be delayed by up to 2 years, which can be a limiting factor in patients with progressive epilepsy. The use of SRT and LITT has been largely influenced by center experience and regional difference in availability of both methods. Unfortunately, prognostication of outcome in all surgical approaches is complicated. Although some studies suggest a correlation with hamartoma size, attachment type, or epilepsy duration, prediction of outcome for individual patients is inconclusive. This is especially critical in a scenario where early surgical intervention is recommended to prevent disease progression.^11^ Most centers using LITT or SRT aim to disconnect the HH from surrounding tissue rather than completely removing/destroying the lesion. Particularly in SRT, selecting surgical trajectories and target volumes has been largely variable depending on the surgical center.^13^, ^14^ The largest reported series has been conducted in Japan, with approximately 240 patients and relatively large interventions that required on average two burr holes, four trajectories, and eight coagulation sites.^15^ Smaller studies report similar outcomes with choosing maximally only two trajectories and six coagulation sites.^16^, ^17^

In this study, we retrospectively report the outcome of SRT conducted at the Epilepsy Center in Freiburg between 2016 and 2024 using an approach explicitly aiming to disconnect and not to destroy the HH. We here report seizure outcomes of SRT in different HH types, required coagulation volumes for seizure control, and tolerability of the procedures.

## 2 MATERIALS AND METHODS

### 2.1 Patients

Patients with HHs who underwent SRT between July 2016 and June 2024 were retrospectively analyzed. Clinical data were taken from patient's electronical medical records. All patients received a standardized presurgical evaluation including medical history, neurological and endocrinological examinations, video‐electroencephalography (EEG) of at least 72 h, magnetic resonance imaging (MRI), and neuropsychological assessment. For every patient, two independent raters determined whether global developmental delay/intellectual disability was present. Postsurgical follow‐up evaluations were conducted at 3 and 6 months, at 1 year, and annually thereafter when possible. Seizure outcomes were defined according to the Engel classification; additionally, seizure outcome for gelastic seizures was noted separately from overall seizure outcome. Any unexpected effect persisting beyond the 12‐month follow‐up was classified as a persistent complication. This retrospective study received approval from the local ethics committee (#400/20); informed consent was not required.

### 2.2 MRI analysis and volumetry

Routine MRI assessment followed the local standard protocol (Table S1), similar to the International League Against Epilepsy recommended HARNESS (Harmonized Neuroimaging of Epilepsy Structural Sequences) MRI protocol.^18^ HHs were categorized according to the Delalande classification based on coronal magnetic resonance images. Additionally, HH attachment was classified as unilateral or bilateral depending on its continuity with the hypothalamus. Hypothalamic hamartoma volumes were segmented from preoperative T1‐weighted (T1w) and T2‐weighted MRI sequences using Smart Brush in Elements software (Brainlab).

### 2.3 Surgical procedure

SRT trajectory planning was typically performed 24 h preoperatively by an interdisciplinary team of neuroradiologists, epileptologists, and neurosurgeons. Planning was based on comprehensive multimodal MRI analysis, including three‐dimensional (3D) T1‐weighted magnetization‐prepared rapid acquisition gradient echo (with and without contrast), 3D fluid‐attenuated inversion recovery (FLAIR) sequence, and 3D magnetization‐prepared two rapid acquisition gradient echoes and after discussion of the video‐EEG monitoring data. Single or multiple trajectories were planned on which the coagulation points were lined up to achieve an optimal disconnection of the hamartoma. Critical emphasis was placed on disconnecting the hamartoma between the fornix and mammillothalamic tract while preserving these anatomical structures. For bilaterally connected hamartomas, the optimal disconnection side was determined through comprehensive multimodal analysis, integrating epileptological data (seizure semiology and EEG monitoring findings) and 3D T1w and FLAIR MRI sequences. Trajectory planning was executed using Leksell SurgiPlan software (Elekta).

All procedures were performed under general intravenous anesthesia using the Leksell Frame G stereotactic system (Elekta). Following frame placement, stereotactic computed tomography angiography (CTA) was acquired using a Somatom Definition AS 64 scanner (Siemens Healthcare) with standardized acquisition parameters (120 kV, 64 × .6 mm spiral acquisition with 1‐mm isotropic reconstruction) after cubital intravenous injection of 50 mL contrast agent (Imeron 400). Scans were started manually with a 2‐s scan delay based on visual bolus tracking at a midcervical level. The CTA dataset was coregistered with preoperative MRI and planned trajectories in Leksell SurgiPlan, allowing trajectory refinement based on vascular anatomy when indicated.

For each trajectory, lesion electrode positioning was verified using a permanently installed stereotactic X‐ray system (Precisis) in the operating room. The system is aligned with the operating table and the mounted stereotactic frame to enable X‐ray beam projection precisely along the axis through both targeting guides of the center‐of‐arc stereotactic system (Leksell Frame G, Elekta). Radiofrequency thermocoagulation was performed through a burr hole using a dedicated electrode (2‐mm diameter, 4‐mm exposed tip) connected to a lesion generator (LG2) and guided by a precision microdrive system (both Inomed). Initial test coagulation was performed at 60°C for 30 s, followed by therapeutic lesioning at 74°C for 60 s after confirmation of stable physiological parameters. Multiple lesions were generated along single trajectories where indicated. The electrode was repositioned only after cooling to sub‐39°C temperatures to ensure tissue integrity. Postoperative monitoring was conducted in the postanesthesia care unit.

### 2.4 Volumetry of coagulation

Volumetric analyses were conducted to quantify the coagulated volumes, their coverage ratio of the hamartoma, and the spatial overlap between individual coagulation zones. Using multimodal imaging data (preoperative MRI: 3D T1w and FLAIR MRI sequences, computed tomography [CT] with stereotactic frame and reference box) and the stereotactic coordinates of trajectories and coagulation points, we modeled spherical coagulation volumes (2.5‐mm radius) for each ablation point using 3D planning software (Elements and GuideXT, Brainlab). The software enabled calculation of the effective total coagulated volume (*T*_coag_). We derived two key metrics: (1) the ratio between total coagulated and hamartoma volumes (*T*_coag_/*V*_HH_), representing overall coverage; and (2) the ratio between the sum of individual coagulation sphere volumes and the total coagulated volume (∑_coag_/*T*_coag_), indicating the degree of overlap between coagulation zones. For the latter metric, a value of 1 indicates no overlap, whereas 2 signifies double coverage of the entire volume.

### 2.5 Statistical analysis

All statistical analyses were conducted using Prism version 10.2.3 (GraphPad Software, www.graphpad.com). Statistical significance was defined as *p* < .05. Data are presented as mean with SD or median with range, as appropriate. Group comparisons were performed using Fisher exact test for categorial variables and Mann–Whitney *U*‐test for continuous variables. Spearman rank correlation coefficient was employed to assess the correlation between different volumetric parameters.

## 3 RESULTS

### 3.1 Patient characteristics

**FIGURE 1:**
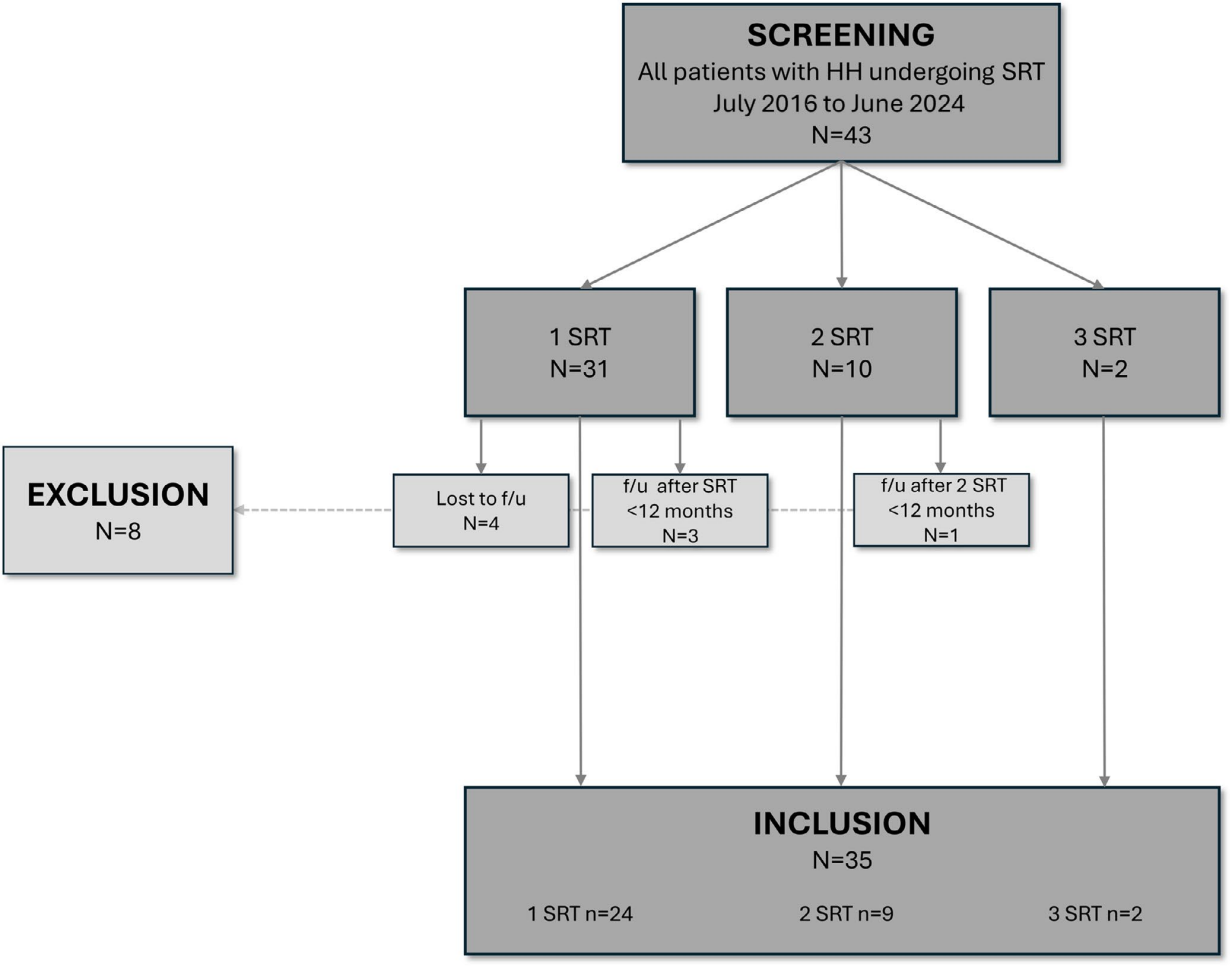
Study flow. f/u, follow‐up; HH, hypothalamic hamartoma; SRT, stereotactic radiofrequency thermocoagulation.

A total of 43 patients with HH underwent SRT between July 2016 and June 2024. Of these, 35 patients with a minimum follow‐up period of 12 months were included in the analysis. The majority of patients (*n* = 24) underwent a single SRT, whereas nine patients underwent two and two patients underwent three SRTs (Figure 1).

The cohort consisted of 22 children and 13 adults, with a median age of 13 years at the first SRT (range = 2–62). The median duration of epilepsy prior to first SRT was 11 years (range = 1–57). Prior interventions were reported in 22.9% of the patients. Bilateral attachment of the hamartoma was observed in eight patients (22.9%). Comorbidities were present in 60% of patients. Detailed patient characteristics are presented in Table 1.

**TABLE 1:** Patient characteristics.

Characteristic	All, *N* = 35	Children, *n* = 22	Adults, *n* = 13
Sex, male, *n* (%)	23 (65.7)	13 (59.1)	10 (77)
Age at first SRT, years, median (range)	13.0 (2.0–62.0)	6.8 (2.0–17.3)	38.3 (23.0–62.0)
Age at epilepsy onset, years, median (range)	1.4 (0–14.4)	1.2 (0–5.4)	3.0 (.3–14.4)
Epilepsy duration at first SRT, years, median (range)	11 (1–57)	5.5 (1–16)	31 (20–57)
ASMs, number discontinued, median (range)	3 (0–17)	2.5 (0–8)	4 (1–17)
ASMs, number at first SRT, median (range)	2 (0–4)	2 (0–4)	2 (1–3)
Previous interventions, *n* (%)			
Any intervention	8 (22.9)	4 (18.2)	4 (30.8)
Open/endoscopic surgery	3 (8.6)	3 (13.6)	0
Radiosurgery; [one/twice/three times]	5 (14.3); [0/4/1]	1 (4.5); [0/1/0]	4 (30.8); [0/3/1]
Delalande stage, *n* (%)			
I	1 (2.9)	0	1 (7.7)
II	21 (60)	12 (54.5)	9 (69.2)
III	5 (14.3)	2 (9.1)	3 (23.1)
IV	8 (22.9)	8 (36.4)	0
Volume of HH, mL			
Mean (SD)	1.86 (3.61)	2.59 (4.40)	.59 (.52)
Median (range)	.46 (.08–16.2)	.49 (.12–16.2)	.44 (.08–1.58)
Side of attachment, *n* (%)			
Right	15 (42.9)	9 (40.9)	6 (46.2)
Left	12 (34.2)	5 (22.7)	7 (53.8)
Bilateral	8 (22.9)	8 (36.4)	0
Seizure types within 3 months prior to first SRT, *n* (%)			
Gelastic	26 (74.3)	18 (81.8)	8 (61.5)
Motor	18 (51.4)	13 (59.1)	5 (38.5)
Nonmotor	17 (48.6)	7 (31.8)	10 (76.9)
Focal to bilateral tonic–clonic	14 (40)	6 (27.3)	8 (61.5)
Epileptic spasms	1 (2.9)	1 (4.5)	0
Gelastic seizures only	2 (5.7)	2 (9.1)	0
One seizure type only	6 (17.1)	5 (22.7)	1 (7.7)
Two seizure types	18 (51.4)	12 (54.5)	6 (46.2)
≥Three seizure types	11 (31.4)	5 (22.7)	6 (46.2)
Seizure frequency, *n* (%)			
Daily	29 (82.9)	21 (95.5)	8 (61.5)
Weekly	6 (17.1)	1 (4.4)	5 (38.5)
Intellectual functioning prior to first SRT, *n* (%)			
Borderline or average	19 (54.3)	10 (45.5)	9 (69.2)
GDD/ID	16 (45.7)	12 (54.5)	4 (30.8)
Comorbidities, *n* (%)			
None	14 (40)	9 (40.9)	5 (38.5)
Precocious puberty	9 (25.7)	6 (27.3)	3 (23.1)
Behavioral	10 (28.6)	8 (36.4)	2 (15.4)
Obesity	6 (17.1)	5 (22.7)	1 (7.7)
Autism spectrum disorder	6 (17.1)	6 (27.3)	0
Endocrine^a^	2 (5.7)	2 (9.1)	0
Pallister–Hall or PTEN hamartoma tumor syndrome	3 (8.6)	3 (13.6)	0
Other^b^	13 (37.1)	6 (27.3)	7 (53.8)

Abbreviations: ASM, antiseizure medication; GDD, global developmental delay; HH, hypothalamic hamartoma; ID, intellectual disability; PTEN, phosphatase and tensin homolog; SRT, stereotactic radiofrequency thermocoagulation.

^a^ Endocrine (*n* = 1 each): hypocortisolism, hypothyroidism.

^b^ Other (*n* = 1 each): children: folate deficiency, Langerhans cell histiocytosis, osteoporosis, tic disorder, vitamin D deficiency, Von Willebrand disease; adults: asthma, chronic hepatitis B virus infection, depression, nephrolithiasis, nonepileptic seizures, sleep apnea.

### 3.2 Seizure outcome

Freedom from all disabling seizures (Engel class I outcome) was achieved in 60.0% of patients at follow‐up 12 months after last SRT, and freedom from bilateral tonic–clonic seizures was achieved in 88.6%. Overall, median follow‐up period after last SRT was 36 months (range = 12–91). Seizure worsening was not observed in any of the patients. All seizure outcomes at 12 months and at last follow‐up are presented in Table 2. The median number of ASMs at last follow‐up was 1 (range = 0–4), representing a significant reduction from baseline (median = 2, range = 0–4, *p* = .034). A reduction in ASM count by ≥1 was observed in 38.1% of patients. Other than a significantly lower median number of ASMs at last follow‐up in children compared to adults (median = 1 [range = 0–4] vs. median = 2 [range = 1–3], *p* = .03), no significant differences in seizure outcome were observed between the two groups.

**TABLE 2:** Seizure outcomes.

Outcome	12 months after last SRT	Last follow‐up^a^
Engel outcome, *n* (%)		
Engel I	21 (60.0)	19 (54.3)
Engel II	3 (8.6)	5 (14.3)
Engel III	6 (17.1)	5 (14.3)
Engel IV	5 (14.3)	6 (17.1)
Free of gelastic seizures, *n* (%)	27 (77.1)	26 (74.3)
Gelastic seizures only, *n* (%)	5 (14.3)	5 (14.3)
Free of bilateral tonic–clonic seizures, *n* (%)	31 (88.6)	31 (88.6)
ASMs, *n*, median (range)	1 (0–4)	1 (0–4)

Abbreviations: ASM, antiseizure medication; SRT, stereotactic radiofrequency thermocoagulation.

^a^ Median follow‐up period after last SRT = 36 months (range = 12–91).

In total, 11 patients (seven children, four adults) underwent more than one SRT. Median time between the first and last SRT was 10 months (range = 5–30). Seizure outcome improved more than one Engel class in five patients (including three patients improving from class III or IV to class I). In the remaining patients, Engel outcome remained the same in four and worsened (from class III to class IV) in two patients.

### 3.3 Adverse effects/surgical complications

Transient and expected treatment‐emergent adverse events primarily presented as contralateral emotional facial paresis (EFP; *n* = 10) and hypothalamic dysfunction, including sleepiness (*n* = 3), syndrome of inappropriate antidiuretic hormone secretion (*n* = 1), combined hypocortisolism and hypothyroidism (*n* = 1), and Horner syndrome (*n* = 4). Additionally, hypotonia/hypokinesia of the contralateral limbs, likely transient supplementary motor symptoms, were observed in four patients and vertical gaze palsy in one patient. Recovery of motor symptoms and hypothalamic dysfunction occurred within 12 h to 3 days, whereas resolution of EFP and Horner syndrome ranged from a few days to several months. Notably, 10 children and two adults experienced transient central fever postsurgery without signs of infection. This occurred in 75% of cases on the first postoperative day and in 25% on the second day (median temperature = 38.8°C, range = 38.6–39.9; median duration = 24 h, range = 6–36). Persistent complications occurred in 10% of interventions (Table 3). A small infarction in the internal capsule was observed in the patient with mild unilateral paresis. Additionally, minor hemorrhages within the HH were detected in the patient with mild verbal memory deficits. Incidental findings on CT revealed small hemorrhages within the HH or along the surgical trajectory in two patients, without any associated symptoms.

**TABLE 3:** Persistent surgical complications.

Complication	Coagulations, *n* = 48, *n* (%)	Patients, *n* = 35, *n* (%)
Any complication	5 (10.4)	5 (14.3)
Hypothalamic dysfunction [all obesity]	3 (6.3)	3 (8.6)
Mild unilateral paresis	1 (2.1)	1 (2.9)
Mild deficits in verbal memory	1 (2.1)	1 (2.1)

### 3.4 Volumetry

**FIGURE 2:**
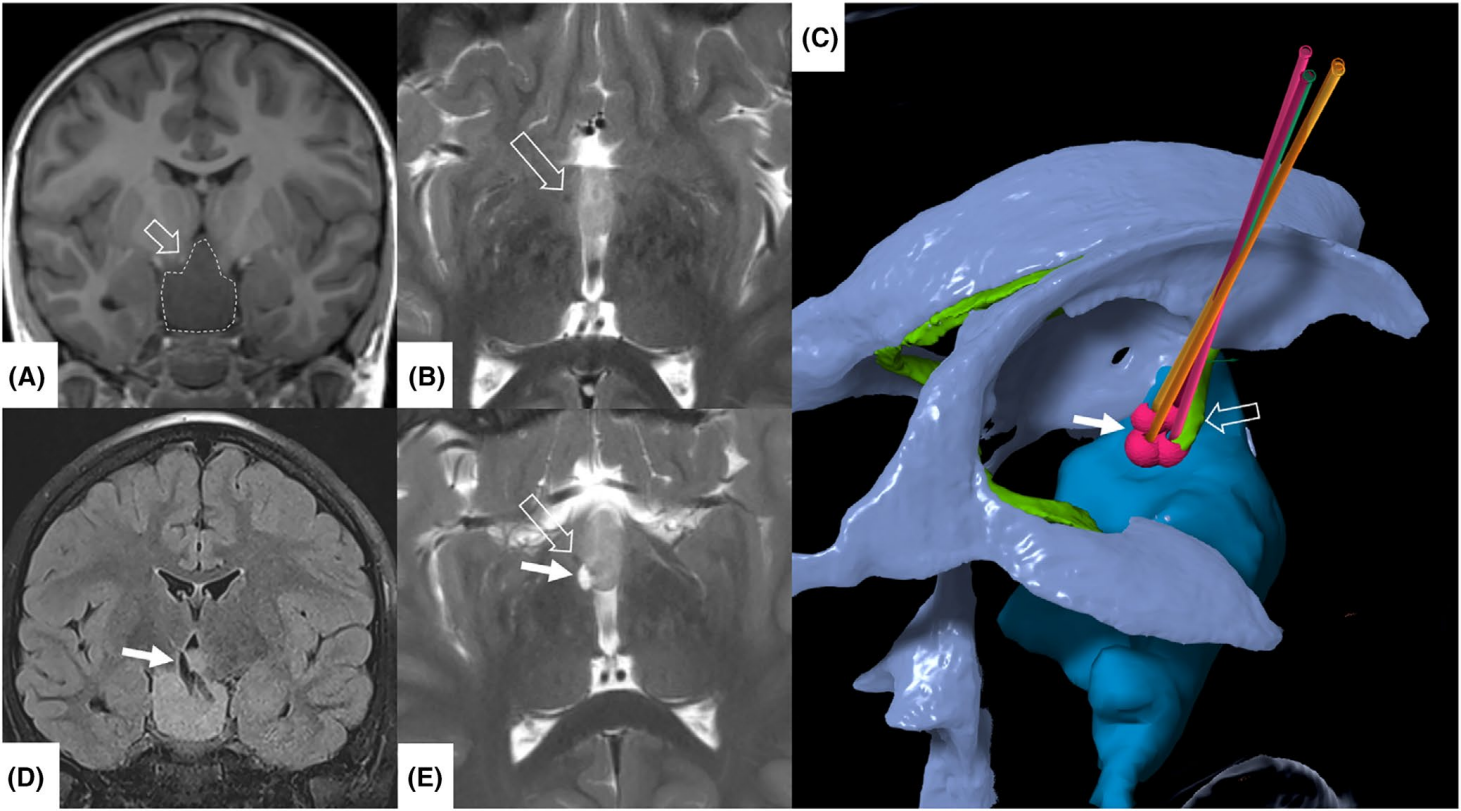
Case study of 6‐year‐old female patient with bilateral, predominantly right‐sided pedunculated hypothalamic hamartoma (HH) with Delalande IV and Engel class I outcome. Preoperative imaging: (A) Coronal T1‐weighted imaging. Dashed margin indicates HH. (B) Axial T2‐weighted imaging. Fornix is marked with an open arrow as an anatomical landmark and risk structure. (C) Three‐dimensional reconstruction of stereotactic planning. Blue: HH; red: planned coagulation volumes along the four planned trajectories; green: fornix; purple: ventricles. (D, E) Postoperative imaging showing the disconnection plane. (D) T2‐weighted fluid‐attenuated inversion recovery coronal view. Coagulation defect is marked with a solid arrow. (E) T2‐weighted axial view. Coagulation defect is marked with a solid arrow, and fornix is marked with an open arrow.

In our cohort, the primary strategy was to disconnect the HH from the hypothalamus. To evaluate the effectiveness of this approach, we assessed several volumetric parameters, including the relationship between the coagulated volume and HH volume, as well as their correlation with seizure outcomes and complication rates. The initial mean *V*_HH_ across the cohort was 1.86 mL (SD = 3.61), and the mean *T*_coag_ during the first SRT was .33 mL (SD = .22). This resulted in a mean ratio of *T*_coag_ to *V*_HH_ of .54 (SD = .4). A strong positive correlation was observed between the initial *V*_HH_ and *T*_coag_ (*R* = .87, *p* = .0001). However, in cases of giant hamartomas, the ratio of *T*_coag_ to *V*_HH_ remained relatively small. For example, in one patient with a *V*_HH_ of 14.1 mL, the *T*_coag_ was only .9 mL (see Figure 2 for more information on this case study).To further assess the intensity of the surgical approach, we calculated the overlap of the coagulation volumes. We therefore calculated the ratio between the sum volume of the single coagulation points (**∑**_coag_) and the de facto *T*_coag_. This overlap coefficient starts at 1.0 (no overlap) and increases with multiple overlapping regions, where values of 2.0, 3.0, or higher indicate double, triple, or greater coverage, respectively. The mean overlap coefficient was 1.43 (.37) for initial SRT procedures and 1.29 (.22) for repeat SRT procedures. Among the 11 patients who underwent a second SRT, both **∑**_coag_ and *T*_coag_ were significantly lower in the second SRT compared to the first. However, no significant differences were observed in the overlap or the ratio of coagulated volume to initial HH volume. All volumetric parameters are detailed in Table 4.

**TABLE 4:** Volumetric parameters.

Parameter	SRT, *n* = 35	SRT, *n* = 11	*p*
**∑**_coag_			.01
Mean (SD)	.46 (.32)	.26 (.14)	
Median (range)	.42 (.07–1.54)	.21 (.07–.56)	
*T*_coag_			.02
Mean (SD)	.33 (.22)	.19 (.10)	
Median (range)	.28 (.07–.99)	.17 (.07–.43)	
Overlap [**∑**_coag_/*T*_coag_]			.16
Mean (SD)	1.43 (.37)	1.29 (.22)	
Median (range)	1.4 (1–3.29)	1.29 (1.0–1.75)	
*T*_coag_/*V*_HH_			.16
Mean (SD)	.54 (.40)	.37 (.24)	
Median (range)	.58 (.06–2.13)	.34 (.09–.89)	

Abbreviations: **∑**_coag_, sum of each coagulation point; HH, hypothalamic hamartoma; SRT, stereotactic radiofrequency thermocoagulation; *T*_coag_, total coagulated volume; *T*_coag_/*V*_HH_ = relation of total coagulated volume to preinterventional HH volume; *V*_HH_, preinterventional HH volume.

We further evaluated whether specific volumetric parameters influenced seizure outcomes or complication rates but did not identify any significant associations. In addition to volumetric parameters, other disease characteristics were analyzed for their impact on seizure outcomes. Factors such as the presence of bilateral tonic–clonic seizures prior to the first SRT, disease duration, Delalande classification, and whether the HH was attached unilaterally or bilaterally also did not show statistically significant differences in relation to outcomes or complications (Table S2).

## 4 DISCUSSION

Our study presents an approach to SRT that differs from reported approaches by using fewer trajectories and fewer coagulation sites to disconnect the HH from the hypothalamus. Overall, seizure freedom rates were slightly lower than reported in patient cohorts undergoing more extensive thermocoagulation, but our approach was highly effective in preventing bilateral tonic–clonic seizures in nearly 90% of all patients and eliminating gelastic seizures in 75% of patients. Our study also provides evidence that seizure freedom can be achieved with small disconnections even in patients with large hamartomas. We did not find a correlation between the overall coagulated volume and the percentage of overall hamartoma tissue that was removed, suggesting that interrupting the connection between hamartoma and hypothalamus is more relevant than volume reduction of the lesion.

### 4.1 Reported surgical outcomes

Recent meta‐analyses suggest that both SRT and LITT achieve comparable success rates in seizure outcomes. Although Iranmehr and coworkers found that SRT was slightly less effective, with 69% of patients achieving seizure freedom compared to 87% with LITT (possibly related to a somewhat shorter median follow‐up in the included LITT studies),^10^ two other studies could not replicate these findings. In these studies, SRT and LITT achieved comparable results, with 72% and 75% seizure freedom rates.^11^, ^12^ Similar discrepancies were observed in complication rates, which were significantly lower for SRT according to one study^10^ and similar according to the other.^11^ All studies, however, consistently reported that radiosurgery was less effective in seizure control, although it had the lowest rate of complications. These variations highlight the inherent difficulties in comparing treatment outcomes for rare conditions like HH, especially given that surgical approaches can vary substantially across centers, even for the same technique. As Rizzi and colleagues emphasize,^11^ center‐specific practices can influence surgical outcomes. Furthermore, the retrospective nature of most meta‐analyses complicates the assessment of outcomes due to heterogeneity in data, including seizure type, follow‐up duration, HH size, and reporting consistency.

In our cohort, seizure outcomes were encouraging despite a minimal intervention approach. With a single SRT procedure in the majority of patients, 60% achieved complete seizure freedom at 1 year. Importantly, seizure control was particularly effective for bilateral tonic–clonic seizures, with >85% of patients experiencing freedom from this seizure type. This allowed for a meaningful reduction in ASM load in many cases. Although early ASM tapering may have influenced long‐term seizure outcomes, it is known to have beneficial effects on cognition and quality of life.^19^ Moreover, even patients who did not achieve complete seizure freedom often experienced significant improvements in seizure severity and frequency, particularly in gelastic and secondary bilateral seizures. Further interventions remain an option and have already been pursued in selected cases, supporting a stepwise and individualized treatment strategy.

Given the nature of Radiofrequency thermocoagulation (RFT) as a minimally invasive technique, repeat interventions are both feasible and often necessary to achieve optimal seizure control. In our study, most patients underwent only one RFT procedure during the study period. However, 30% underwent a second or third intervention, which is comparable to literature reports, which cite repeat procedures in 25%–50% of patients.^15^, ^20^ Notably, surgical success rates following subsequent interventions were similar to those observed after the initial procedure, with comparable rates of seizure freedom. It is important to note that all meta‐analyses as well as several single‐center analyses primarily focus on reporting seizure reduction and seizure freedom.^15^, ^17^, ^20^ In contrast, there is limited information available on the risk of seizure worsening following RFT, as the Engel classification groups seizure reduction of <50% and seizure worsening into class IV, potentially obscuring true deterioration. A few studies on LITT for HH have reported transient or permanent seizure worsening.^21^ In most of these cases, seizure aggravation was managed with a repeat intervention after a period of 4–6 months, during which spontaneous improvement was sometimes observed. In our cohort, none of six patients with Engle outcome class IV experienced actual seizure worsening; rather, they showed limited improvement. Among those, three became seizure‐free after a second procedure. There have been reports of secondary epileptogenesis and progressive epilepsy in patients with HH over time.^22^ In our cohort, which included more children than adults, no correlation was found of epilepsy duration, age at onset, and seizure type with surgical seizure outcome. RFT is a safe method for children as soon as their cranium is stable enough for a frame‐based approach, and no correlation between complication rates and age could be found either.

The majority of treatment‐emergent adverse effects observed after RFT in our cohort were transient, and rates of EFP and Horner syndrome were lower than previously reported.^13^, ^14^ It is important to note that in contrast to LITT interventions, standard application of systemic steroids after the intervention is not necessary in RFT, but temporary tissue swelling can cause transient symptoms. Permanent complications in our cohort were mostly mild. Hypothalamic dysfunction was most common, at approximately 10%, similar to reports in the literature.^11^ Especially, hypothalamic obesity and diabetes insipidus can be permanent and disabling. These complications were not correlated with the volume coagulated during the intervention and not reduced compared to RFT approaches from the literature that use more trajectories or coagulation points. All patients at our center received standardized neuropsychological testing and neurological exams, which is recommended but not mandatory.^6^, ^23^ In our study, we considered the transition from temporary to permanent complication when symptoms persisted for >12 months. Some of the larger series on RFT and complications did not clarify this separation and considered improvements possible after 12 months of recovery.^13^ It is therefore difficult to provide a direct comparison of complications between different studies, but our rates for temporary and permanent deficits seem similar to those reported in other studies. Although we would have expected that the use of fewer trajectories and coagulation sides would not only result in fewer transient adverse effects but also result in less persistent complications, this is not supported by our current results.

### 4.2 Disconnection versus volume reduction

A key technical difference between RFT and LITT is that LITT allows near real‐time magnetic resonance thermal imaging, enabling dynamic monitoring and estimation of the ablation zone during the procedure. In contrast, RFT relies entirely on preoperative planning, and the extent of the coagulation cannot be adapted during the procedure. This limitation may lead to smaller coagulation volumes and a more conservative approach in RFT, which could partially explain the differences in seizure freedom rates found in some meta‐analyses.^10^ Gadgil and colleagues performed a detailed analysis of the trajectories used and volume reductions archived with LITT.^21^ Interestingly, their data showed that the actual volume of the ablation was smaller on the postoperative MRI 3 months after the intervention than predicted according to the intraoperative imaging. Their data also suggested that a reduction of the hamartoma volume by approximately 50% is correlated with better seizure outcomes, but also that a complete disconnection of the HH is more important than a complete removal. Although our data also suggested that the coagulated volume was higher in larger HH, we did not find any correlation between coagulation volume and surgical outcomes. Even very large lesions resulted in seizure‐free outcomes after achieving complete disconnections. The main challenge for the future will be to identify the most important connections from the HH to other brain regions, especially for those lesions that are embedded in the hypothalamus.

Some RFT methods are based on using stereo‐EEG electrodes. These allow recording neurophysiological activity, including epileptic activity, from the hypothalamic tissue and hamartoma prior to the coagulation. Using this approach, one might be able to find a border between physiological tissue in the hypothalamus and abnormal hamartoma cells.^24^ Although stereo‐EEG (SEEG) is not generally considered useful in the presurgical evaluation of HH,^6^ strategically placed electrodes might allow identification of areas of seizure onset and propagation during the RFT procedure.^20^, ^25^ In our study, thermocoagulation was not conducted via SEEG electrodes, but an independent coagulation probe was inserted, guided by a stereotactic frame system, limiting the ability to adjust the disconnection according to EEG activity. Evidence that surgical outcomes can be improved using interictal or ictal activity at the border of a disconnection is currently limited, and prospective trials would be needed to address this question. Endoscopic surgery, which was initially used for resection, is now increasingly applied with the goal of disconnection. An advantage of this technique is that it allows direct visualization of the HH attachment to the wall of the third ventricle, enabling real‐time control of the resection or disconnection. However, the underlying surgical strategy—resection versus disconnection—is often not clearly described in the published series, making it difficult to assess the true efficacy of this method. Nevertheless, valuable lessons can be drawn from these experiences; for instance, whether a transventricular approach may offer specific benefits warrants further investigation.^11^, ^12^ Another suggested approach to tailor disconnected surgeries has been the use of pre‐ and intraoperative connectivity analyses as part of planning and conducting LITT procedures. Boerwinkle and colleagues demonstrated that integrating resting state functional MRI (fMRI) into surgical planning can improve seizure outcomes in HH.^26^ Using connectivity analysis, this group increased seizure freedom rates after LITT intervention by nearly 20% compared to a historical cohort. Another study used fMRI connectivity analysis to compare pre‐ and postoperative networks during the LITT procedure. Seizure‐free patients showed significant changes in their postoperative compared to preoperative networks, in contrast to patients who continued to have seizures. Whether fMRI and connectivity analysis will help us to tailor resections in the future and whether intraoperative MRI is needed for these benefits is not completely clear at the moment. Several studies, however, suggest that bilateral attachment of hamartomas is a negative predictor, both for seizure outcome and complication rates. Thus, methods that allow us to understand propagation pathways between the HH and the cortex are likely to improve surgical precision and outcomes.^27^, ^28^

The principle of achieving effective disconnection through strategic targeting rather than extensive ablation is further supported by single‐trajectory approaches utilizing intraventricular visualization. Robotic stereo‐endoscopic techniques allow direct visualization of the HH attachment to the hypothalamic wall, enabling precise placement of multiple laser or radiofrequency applications through a single burr hole and trajectory.^7^, ^8^, ^9^ This approach exemplifies how different technical solutions can achieve the same therapeutic goal: strategic disconnection of critical epileptogenic connections while minimizing tissue damage. Whether achieved through our frame‐based stereotactic approach with limited trajectories, or through single‐trajectory intraventricular techniques, the underlying principle remains consistent; identifying and disconnecting the crucial epileptogenic interface between the HH and hypothalamus is more important than achieving extensive volume reduction.^7^, ^8^ These various approaches demonstrate that the choice of technique should be guided by hamartoma anatomy, surgeon expertise, and available technology, while maintaining focus on the fundamental goal of effective disconnection.

In the past, with open or endoscopic surgical techniques aimed at resecting the HH, larger HH size was often associated with a poorer outcome.^12^ Several studies using SRT or LITT, including our own, have found that HH size, categorized by the Delalande classification, does not strongly correlate with seizure outcome.^13^ Older classification systems, including the widely used Delalande classification, have traditionally shown good correlation with clinical data. However, with the advent of new treatment options, the attachment pattern of the HH has become increasingly relevant for surgical planning. Based on this consideration, Shirozu and coworkers have proposed a new classification system, which categorized hamartomas according to their attachment into the following groups: parahypothalamic–unilateral, parahypothalamic–bilateral, intrahypothalamic–unilateral, intrahypothalamic–bilateral, mixed–unilateral, and mixed–bilateral types.^15^ Application of the new classification to the large Japanese RFT cohort of 218 patients suggests that the attachment type correlates with the number of trajectories needed to treat the HH successfully and complication rates. This interesting hypothesis needs confirmation in future patient series.

## 5 CONCLUSIONS

Here, we report data on a single‐center series using SRT to treat HH in patients with refractory epilepsy and their long‐term outcome. Detailed analysis of volumetric data suggests that outcome is not affected by size reduction and volume coagulated by rather by effective disconnection of the hamartoma. We also demonstrate that strategic disconnection using limited trajectories and coagulation sites can successfully treat seizures and was especially successful in stopping bilateral tonic–clonic seizures, a principle that can be achieved through various technical approaches including frame‐based stereotactic methods and single‐trajectory intraventricular techniques. More prospective studies will be needed to better understand which degree of disconnection is needed to predict seizure freedom and which diagnostic tools such as functional imaging and EEG could be used to facilitate tailoring of targets.

## AUTHOR CONTRIBUTIONS

**Peter Christoph Reinacher, Julia Jacobs, Kerstin Alexandra Klotz:** Conceptualization; methodology; investigation; data curation; formal analysis; writing—original draft; writing—review & editing. **Andreas Schulze‐Bonhage:** Conceptualization; writing—original draft; writing—review & editing. **Theo Demerath, Kathrin Wagner, Victoria San Antonio‐Arce:** Conceptualization; investigation; writing—review & editing. **Mukesch Johannes Shah, Horst Urbach, Volker Arnd Coenen:** Writing—review & editing.

## CONFLICT OF INTEREST STATEMENT

P.C.R. has received research support from the Else Kröner Fresenius Foundation, Fraunhofer Foundation (ATTRACT), German Ministry for Economic Affairs and Energy, and Medical Faculty of the University of Freiburg. He has received personal honoraria for lectures or advice from Boston Scientific, Brainlab, Inomed, and Fraunhofer Foundation and is a consultant to Boston Scientific, Brainlab, and Inomed. T.D. is a consultant for Medtronic and has received travel/educational grants from Balt, Stryker, and Medtronic. H.U. has received honoraria for lectures from Bayer, Biogen, GE, Eisai, Mbits, and Lilly, is supported by the German Federal Ministry of Education and Research, and is coeditor of *Clinical Neuroradiology*. V.A.C. has received a collaborative grant from Brainlab (Munich, Germany). He is a consultant for Ceregate (Munich, Germany) and Cortec (Freiburg, Germany). He is involved in an ongoing investigator‐initiated trial with Boston Scientific (USA) and has received personal honoraria and travel support for lecture work from Boston Scientific (USA). None of the other authors has any conflict of interest to disclose. We confirm that we have read the Journal's position on issues involved in ethical publication and affirm that this report is consistent with those guidelines.

## Supporting information


DATA S1

## Data Availability

The data that support the findings of this study are available from the corresponding author upon reasonable request.
