# Supplementary material for: Long‐term outcomes of stereotactic radiofrequency ablation in hypothalamic hamartomas: A single‐center experience
Source: Epilepsia. 2025 Oct 1;67(1):96–108. doi: 10.1111/epi.18660 (PMC12893250; doi:10.1111/epi.18660)
Supplement: Supplementary file 1 — DATA S1 [file EPI-67-96-s001.docx]

| **MRI sequence** | **No. of slices/thickness (mm)** | **Voxel size (mm^3^)** | **TI/TR/TE/α (ms/ms/ms/°)** | **Acquisition time (min:s)** |
| --- | --- | --- | --- | --- |
| Sag 3D MPRAGE | 160/1 | 1 × 1 × 1 | 900/2000/2.26/12 | 4:40 |
| Sag 3D FLAIR-SPACE | 160/1 | 1 × 1 × 1 | 1800/5000/388/var | 6:52 |
| Ax 2D T2-TSE | 42/3 | 0.4 × 0.4 × 3 | 5040/102/150 | 4:34 |
| Ax 2D T2* | 23/5 | 0.7 × 0.7 × 5 | 639/19.9/20 | 2:33 |
| Cor 2D T2-STIR | 40/2 | 0.4 × 0.4 × 2 | 100/5390/25/140 | 8:07 |
| Cor 2D FLAIR | 68/2 | 0.7 × 0.7 × 2 | 2500/9000/87/150 | 4:14 |
| Ax 2D DWI-SE EPI | 23/5 | 0.6 × 0.6 × 5 | 3400/85 | 0:46 |
| Sag 3D MP2RAGE | 192/1 | 1 × 1 × 1 | 700, 5000/2000/2.9/4 | 8:52 |

**Table S1:** MRI protocol

*MPRAGE* Magnetization Prepared Rapid Gradient Echo, *FLAIR SPACE* Fluid-Attenuated Inversion Recovery—Sampling Perfection with Application-optimized Contrasts by using flip angle Evolution, *TSE* Turbo Spin Echo, *STIR* Short Tau Inversion Recovery, *DWI* Diffusion-Weighted Imaging, *SE* Spin Echo, *EPI* Echo Planar Imaging, *TI* inversion time, *TR* repetition time, *TE* echo time, *α* flip angle, *var* variable flip angle, *Sag* sagittal, *Ax* axial, *Cor* coronar, *T2** T2 gradient echo

**Supplement Table S2** Univariate analysis of factors influencing seizure outcome or complication rate

|  | **Seizure outcome** | |  |
| --- | --- | --- | --- |
|  | Engel I (n=15) | Engel II-IV (n=20) | P value |
| V_HH_  Mean (SD)  Median (range) | 1.88 (3.72)  0.43 (0.12-14.1) | 1.79 (3.64)  0.48 (0.08-16.2) | 0.79 |
| T_coag_ / V_HH_  Mean (SD)  Median (range) | 0.56 (0.35)  0.60 (0.06-1.44) | 0.53 (0.45)  0.49 (0.06-2.13) | 0.65 |
| Overlap  Mean (SD)  Median (range) | 1.40 (0.18)  1.40 (1.0-0.75) | 1.45 (0.47)  1.38 (1.0-3.29) | 0.57 |
| Delalande n (%)  I  II  III  IV | 0  9 (60.0)  2 (13.3)  4 (26.7) | 1 (5.0)  12 (60.0)  3 (15.0)  4 (20.0) | 0.86 |
| Attachment n (%)  Unilateral  bilateral | 12 (80.0)  3 (20.0) | 16 (80.0)  4 (20.0) | 1.0 |
| Disease duration [years]  Mean (SD)  Median (range) | 15.34 (16.23)  10.5 (1-57) | 17.0 (14.4)  12.5 (2-49) | 0.74 |
| Bilateral tonic clonic seizures pre-SRT n (%) | 6 (40.0) | 8 (40.0) | 1.0 |
|  | **Complication rate** | |  |
|  | No permanent complications (n=27) | Permanent complications (n=8) | P value |
| V_HH_  Mean (SD)  Median (range) | 2.03 (4.00)  0.46 (0.08-16.2) | 1.27 (1.86)  0.49 (0.33-5.80) | 0.75 |
| T_coag_ / V_HH_  Mean (SD)  Median (range) | 0.33 (0.22)  0.29 (0.07-1.0) | 0.32 (0.24)  0.25 (0.11-0.88) | 0.69 |
| Overlap  Mean (SD)  Median (range) | 1.38 (0.17)  1.4 (1.0-0.70) | 1.59 (0.71)  1.35 (1.17-3.29) | 0.79 |

Abbreviations: SRT=stereotactic radiofrequency thermocoagulation; V_HH_=pre-interventional HH-Volume; **∑**_coag_=sum of each coagulation point; T_coag_=total coagulated volume; T_coag_ / V_HH_=Relation of total coagulated volume to pre-interventional HH-Volume; Overlap=**∑**_coag_ / T_coag_
